# The Effects of 10 Hz and 20 Hz tACS in Network Integration and Segregation in Chronic Stroke: A Graph Theoretical fMRI Study

**DOI:** 10.3390/brainsci11030377

**Published:** 2021-03-16

**Authors:** Cheng Chen, Kai Yuan, Winnie Chiu-wing Chu, Raymond Kai-yu Tong

**Affiliations:** 1 Department of Biomedical Engineering, The Chinese University of Hong Kong, Hong Kong 999077, China; chen_cheng@link.cuhk.edu.hk (C.C.); kaiyuan@link.cuhk.edu.hk (K.Y.); 2 Department of Imaging and Interventional Radiology, The Chinese University of Hong Kong, Hong Kong 999077, China; winniechu@cuhk.edu.hk

**Keywords:** transcranial alternating current stimulation, chronic stroke, functional magnetic resonance imaging, graph theory, segregation and integration of brain networks

## Abstract

Transcranial alternating current stimulation (tACS) has emerged as a promising technique to non-invasively modulate the endogenous oscillations in the human brain. Despite its clinical potential to be applied in routine rehabilitation therapies, the underlying modulation mechanism has not been thoroughly understood, especially for patients with neurological disorders, including stroke. In this study, we aimed to investigate the frequency-specific stimulation effect of tACS in chronic stroke. Thirteen chronic stroke patients underwent tACS intervention, while resting-state functional magnetic resonance imaging (fMRI) data were collected under various frequencies (sham, 10 Hz and 20 Hz). The graph theoretical analysis indicated that 20 Hz tACS might facilitate local segregation in motor-related regions and global integration at the whole-brain level. However, 10 Hz was only observed to increase the segregation from whole-brain level. Additionally, it is also observed that, for the network in motor-related regions, the nodal clustering characteristic was decreased after 10 Hz tACS, but increased after 20 Hz tACS. Taken together, our results suggested that tACS in various frequencies might induce heterogeneous modulation effects in lesioned brains. Specifically, 20 Hz tACS might induce more modulation effects, especially in motor-related regions, and they have the potential to be applied in rehabilitation therapies to facilitate neuromodulation. Our findings might shed light on the mechanism of neural responses to tACS and facilitate effectively designing stimulation protocols with tACS in stroke in the future.

## 1. Introduction

Nowadays, stroke is the leading cause of death worldwide, and survivors undergo different dysfunctions, especially in the motor aspect [1]. Hence, it is essential for stroke subjects to restore functional abilities in order to diminish the inconvenience in daily-living activities. The existence of neuroplasticity, which is an intrinsic property of the human brain to change its function and reorganize after a lesion forms, makes this possible [2]. Meanwhile, there have been various rehabilitation strategies proposed, including conventional physical therapies as well as advanced robot-assisted methods [3,4]. Except for these therapies that influence brain reorganization in a round-about way, the transcranial current stimulation (tCS), which non-invasively modulates the activity of the brain, has attracted increasing attention [5].

Among many available tCS techniques, transcranial direct current stimulation (tDCS) and transcranial alternating current stimulation (tACS) are two typical methods that have intrigued the researchers in the field of neuroscience. The core difference between these two simulations is the form of the currents elicited. In tDCS, a direct current flows from anodal to cathodal electrodes. The effect of tDCS is often related to membrane depolarization, which leads to an increase of the excitation in neurons underneath anodal electrode, but the inhibition of neurons under cathodal electrode [6,7]. When compared with tDCS, tACS has not been thoroughly investigated due to its potentially complicated mechanism and interfering with inherent frequency-specific oscillations in the human brain, let alone the effects in patients with neurological disorders, including stroke. While the underlying neurophysiological mechanism is unknown, the stimulation effect is often attributed to the manipulation and entrainment of intrinsic oscillations in the brain [8]. In the human brain, the communication within and between brain regions was facilitated by synchronized oscillatory activities and the components with various frequencies playing different roles in functioning [9]. This implied that the stimulation effect of tACS might differ, depending on the eliciting frequency of the alternating current. At the same time, it has been indicated that the activity of alpha (8–12 Hz) and beta (13–30 Hz) frequency is prominent in the sensorimotor cortex in the resting-state [10]. Therefore, in our study, because the tACS was imposed on the primary motor area (M1), 10 Hz and 20 Hz as representative alpha and beta stimulating frequencies, respectively, were adopted. Some previous studies have investigated the stimulation effect of 10 Hz and 20 Hz on M1 in healthy subject. It was observed that beta-tACS could be used to induce neurophysiologically detectable state-dependent enhancement effects [11]. 10 Hz and 20 Hz tACS could both facilitate motor sequence learning during a serial reaction time task (SRTT). Additionally, 20 Hz tACS could further stabilize motor control to retain the initial learning rate under interference [12]. Because of the existence of frequency difference, it is reasonable to expect differential effects and a recent study has suggested that this effect exists not only in motor behavior, but also in M1 excitability [13]. However, the tACS studies on stroke subjects were quite scarce, which implied that further investigation is needed.

It is observed that the effects of tCS are not restricted to the stimulated sites, and it also has an impact on the brain network [14]. Besides, for tACS, if the stimulation frequency matches the endogenous oscillation frequency, more pronounced oscillatory effect could be found at the cortical network level [15]. Hence, it is meaningful to explore the stimulation effect of tACS from the brain network perspective. To investigate the brain networks, functional magnetic resonance imaging (fMRI), especially resting-state fMRI, which measures the blood oxygen level (BOLD) signal of different regions in the resting-state, has been widely utilized [16,17]. On the other hand, the graph theory approach could provide an efficient perspective to model and understand the information of integration and segregation property, as well as regional communication in complex networks. Hereby, graph theory could be applied to brain network that is derived from fMRI to provide a framework to evaluate the properties of the constructed network, which has been generally adopted in human neuroscience [18].

Similar to tDCS, which has been applied in rehabilitation therapies [19], tACS could also be a powerful auxiliary tool added to existing therapies. However, before widely utilizing tACS, it is of considerale significance to understand the underlying mechanism of how tACS influences the patterns of patient’s brain. The current study aims to thoroughly explore the frequency-specific stimulation effect of tACS on chronic stroke subjects using graph theory analysis in resting-state fMRI and investigated the modulation effect in motor-related cortical regions. At the same time, the integration and segregation characteristics of the network at the whole-brain level were also investigated. We hypothesized the potential differential effect of 10 Hz and 20 Hz tACS as well as the resulting modulation difference in brain networks in motor-related regions and at the whole-brain level.

## 2. Materials and Methods

### 2.1. Subjects

Thirteen chronic stroke patients (eight males, mean age = 61 ± 10 years) with the right (*n* = 7) or left (*n* = 6) hemisphere impairment were recruited from the local community. The inclusion criteria were: (1) first-ever stroke, (2) sufficient cognitive function to understand instructions (Montreal Cognitive Assessment, Moca score ≥ 22), (3) a single unilateral brain lesion, and (4) more than six months before the experiment. The exclusion criteria were: (1) history of alcohol, drug abuse, or epilepsy, (2) severe cognitive deficits, and (3) any contraindication to tACS or MRI. Fugl-Meyer Assessment for upper-extremity (FMA) and Action Research Arm Test (ARAT) were utilized to assess the motor function of the paretic upper limbs for all stroke patients. The lesion map, detailing demographics and clinical properties of the participants, could be found in the Supplementary Materials (Figure S1 and Table S1). This study was approved by the Joint Chinese University of Hong Kong-New Territories East Cluster Clinical Research Ethics Committee. This study was registered at https://clinicaltrials.gov (accessed on 13 February 2021) (NCT04638192). All of the subjects gave written consent before the intervention.

### 2.2. tACS Intervention

**Figure 1:**
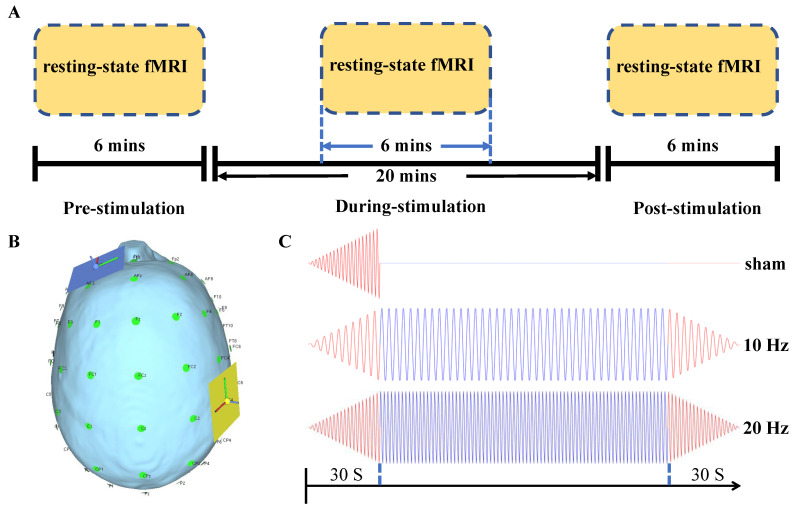
(**A**) The protocol of MRI acquisition and transcranial alternating current stimulation (tACS) intervention. (**B**) The montage of stimulation electrodes (drawn by SimNIBS [22]). The yellow one was put on the ipsilesional primary motor cortex, and the blue one is on the contralesional supraorbital ridge. (**C**) The currents of sham, 10 Hz, and 20 Hz.

According to the international 10–20 system, one electrode (5 × 5 cm${}^{2}$) was positioned over the ipsilesional M1, while the return one was placed over the contralesional supraorbital ridge (Figure 1B). Both of the electrodes were fixed to the patient’s scalp with straps before MRI scanning. For 10 Hz and 20 Hz tACS, an MRI compatible DC-stimulator (NeuroConn GmbH, Ilmenau, Germany) was utilized to deliver the current with 1-mA peak-to-peak intensity for 20 min. The 30-s ramp-up and ramp-down periods at the beginning and end of stimulation, respectively, were adopted. For the sham group, the stimulator was switched off after the 30-s ramp-up period to induce typical tingling sensation [20] (Figure 1C). Each subject would undergo these three stimulation protocols in a randomized order. Meanwhile, the stimulation conditions were performed with a wash-off period of at least one week between each other [21].

### 2.3. Image Acquisition and Preprocessing

A 3T Philips MR scanner (Achieva TX, Philips Medical System, Best, The Netherlands) with an eight-channel head coil was used to acquire high resolution T1-weighted anatomical images (TR/TE = 7.47/3.45 ms, flip angle = 8°, 308 slices, voxel size = 0.6 × 1.042 × 1.042 mm${}^{3}$) while using a T1-TFE sequence (ultrafast spoiled gradient echo pulse sequence), and BOLD fMRI images (TR/TE = 2000/30 ms, flip angle = 70°, 37 slices/volume, voxel size = 2.8 × 2.8 × 3.5 mm${}^{3}$) using a GE-EPI sequence (gradient-echo echo-planar-imaging sequence). Resting-state fMRI data were acquired before, during, and immediately after stimulation. Each run lasted for 6 min. with 180 volumes for each fMRI image (Figure 1A). During acquisition, the patient was instructed to keep awake while focusing on a white cross presented in black background.

The fMRI data were mainly preprocessed using DPARSF toolbox [23]. The first four volumes were removed to assure the remaining volumes of fMRI were at magnetization steady state. Subsequently, the remaining volumes were corrected with slice timing and realigned to correct head motion. Nuisance variables, including white matter, cerebrospinal fluid (CSF), global mean signal, and Friston 24 head motion parameters, were then regressed out [24]. To further control for head motion, the scrubbing process were performed for the volumes with framewise displacement (FD) value exceeding 0.3 [25]. If over 25% of all the volumes exceed the threshold, the corresponding data would be discarded, and no data were discarded in our study. Afterward, the fMRI data were aligned to anatomical images. To remove higher frequency physiological noise and lower frequency scanner drift, detrending, and the 0.01–0.1 Hz band-pass temporal filtering was performed [26]. Subsequently, the functional images were normalized to the Montreal Neurological Institute (MNI) template, resliced to 2 × 2 × 2 mm${}^{3}$ voxels, and then spatially smoothed with a Gaussian kernel with a full-width at half-maximum (FWHM) of 6 mm. The fMRI data of subjects who had left-hemispheric lesions were flipped along the midsagittal plane using MRIcron (www.mccauslandcenter.sc.edu/mricro/mricron (accessed on 13 February 2021)) for group statistical analysis, so that the lesions of all subjects were in the right hemisphere.

### 2.4. Graph Theory Analysis

#### 2.4.1. Construction of Brain Functional Networks

In order to investigate the modification of brain functioning induced by tACS located at the lesioned motor area, the whole brain was first parcelled into 116 regions based on Automated Anatomical Labeling (AAL) atlas [27] to construct the network at the whole-brain level, and 20 regions of interest (ROIs) related to motor function based on previous studies [28] were extracted to constitute the nodes of the network in motor-related regions (Listed in the Supplementary Materials Table S2). The mean time series of each ROI was averaged. The temporal correlation matrix for each subject under each condition was obtained by calculating the Pearson correlation coefficients between the time courses of each pair of regions.

In graph theory, an adjacency matrix was often adopted to characterize the structure of the graph. In the present study, we would threshold the fMRI temporal correlation matrix to acquire group adjacency matrix as well as individual adjacency matrix for each time point under each stimulation condition. First of all, a Fisher’s r-to-z transform was utilized to map correlation r value to z score value for all individual correlation matrices to improve normality [29]. A two-tailed one-sample *t*-test was then used to test the significance of the correlation different from zero for each possible pair of nodes across subjects. A significant level of p<0.01 with Bonferroni correction was adopted to threshold the temporal correlation matrices to obtain the binarized group adjacency matrices. The ratio of the number of existing edges and the maximum number of all edges derived from the resulting group adjacency matrix were used to binarize individual temporal correlation matrix in a proportional-threshold way [30]. Therefore, the inherent structural property of individual adjacency matrix could be consistent with the group adjacency matrix to maximally reduce the bias that is caused by selecting a priori thresholding parameter [31].

#### 2.4.2. Graph Theoretical Measures

After constructions of brain networks, several measures that characterize the property of modular organization and nodes were evaluated. All graph theory analysis was conducted while using Brain Connectivity Toolbox [32] thta was implemented in MATLAB (The MathWorks Inc., Natick, MA, USA).

Modularity. It is often assumed that a brain network always works with several well-partitioned modules or communities, and each community is responsible for specialized functional processing. Modularity is to measure such goodness of graph partitioning, which is defined as [33]:

(1) $$Q=\sum_{u\in M}\left[e_{uu}-{\left(\sum_{v\in M}e_{uv}\right)}^{2}\right]$$

where *u* and *v* represent the specific modules in the set of all subdivided non-overlapping modules *M*, $e_{uv}$ represents the proportion of all links connecting nodes in module *u* and *v*, respectively. *Q* is normally treated as an objective function to the maximize the number of within-module links and minimize the number of inter-module links to optimally subdivide the graph into communities.

Within-module degree z-score. Based on the community assignment of all nodes, the role of a specific node could be determined with respect to its own community as well as other communities. The within-module degree z-score is a classical measure to characterize how ‘well-connected’ a specific node is to other nodes that belong to the same community. Normally speaking, a high value of within-module z-score indicates dense within-module linking [34]. It is defined as:

(2) $$z_{i}=\frac{k_{i}\left(m_{i}\right)-\bar{k}\left(m_{i}\right)}{\sigma^{k(m_{i})}}$$

where $k_{i}$ represents the degree of node *i*, which is equal to the number of links connected to node *i* in the whole network, $k_{i}\left(m_{i}\right)$ represents the number of links between node i and other nodes in the same module, and $\bar{k}\left(m_{i}\right)$ and $\sigma^{k(m_{i})}$ represent the mean and standard deviation of degree distribution in the same module, respectively.

Participation coefficient. Some of the nodes might not merely connect with nodes near them within the same community, but also have connections with nodes in other communities. The participation coefficient is used to evaluate such diversity of inter-modular interconnection for an individual node. Complementary to within-module degree z-score, the participation coefficient characterizes ‘how-distributed’ the links of a specific node among various communities [35], which is defined as:

(3) $$P_{i}=1-{\sum_{m\in M}\left(\frac{k_{i}\left(m\right)}{k_{i}}\right)}^{2}$$

where $k_{i}\left(m\right)$ represents the number of links connecting node *i* and all other nodes in module *m*. It is noted that, if almost all links of a node are restricted within its own community, the participation coefficient of this node is close to 0. Otherwise, the participation coefficient of the node with almost uniformly distributed links tends to be 1.

Clustering coefficient. The clustering coefficient is a kind of measure of segregation that is related to the number of triangles in the network. The nodal clustering coefficient is equivalent to the fraction of the node’s neighbors that are also neighbors of each other, which is defined as [36]:

(4) $$C_{i}=\frac{2t_{i}}{k_{i}\left(k_{i}-1\right)}$$

where $t_{i}=\frac{1}{2}\sum_{j,h\in N}a_{ij}a_{ih}a_{jh}$ ($a_{ij}$ indicates the link between node *i* and node *j*, and *N* means the set of all nodes in the network) represents the number of triangles surrounding node *i*.

Local efficiency. Local efficiency is a nodal measure to characterize the efficiency of local information transmission and mainly focus on the property of communication among neighbors for a specific node [37]. It is defined as:

(5) $$LE_{i}=\frac{\sum_{j,h\in N,j\neq h}a_{ij}a_{ih}{\left[d_{jh}\left(N_{i}\right)\right]}^{-1}}{k_{i}\left(k_{i}-1\right)}$$

where $d_{jh}\left(N_{i}\right)$ indicates the shortest path length of node *j* and node *h*, which contains the only neighbor of node *i*.

Specifically, since we would like to investigate the modulation effect of tACS in motor-related brain regions, we mainly focused on the analysis of the distribution of nodal metrics. The corresponding measure for a node would be calculated by averaging all values across all subjects. The nodal measures during and after stimulation were baseline corrected by subtracting the corresponding values that were derived from the pre-stimulation session to characterize the modulation effect. Besides, the integration and segregation characteristics of the network at the whole-brain level were also investigated.

### 2.5. Statistical Analysis

The statistical tests were conducted using SPSS 25 (IBM SPSS, Armonk, NY, USA). A two-way repeated-measure Analysis of Variance (ANOVA) with factors of stimulation (sham, 10 Hz, and 20 Hz) and time (during and post) was carried out in order to investigate the change of distributions of graph theoretically nodal measures, including within-module degree z-score, participation coefficient, clustering coefficient, and local efficiency. The Greenhouse–Geisser correction would be adopted if Mauchly’s test of sphericity was significant. Paired *t*-tests were applied as *post-hoc* tests to examine whether there exists significant difference in different combinations of three stimulation conditions for each time point. The significance level was set at p< 0.05. Bonferroni correction was used to counteract the problem of multiple comparisons.

## 3. Results

### 3.1. Community Structure

First of all, we investigated the modulation effect of different stimulation protocols that were imposed on the structure of the network in motor-related regions, which is related to the community assignment and affiliation.

**Figure 2:**
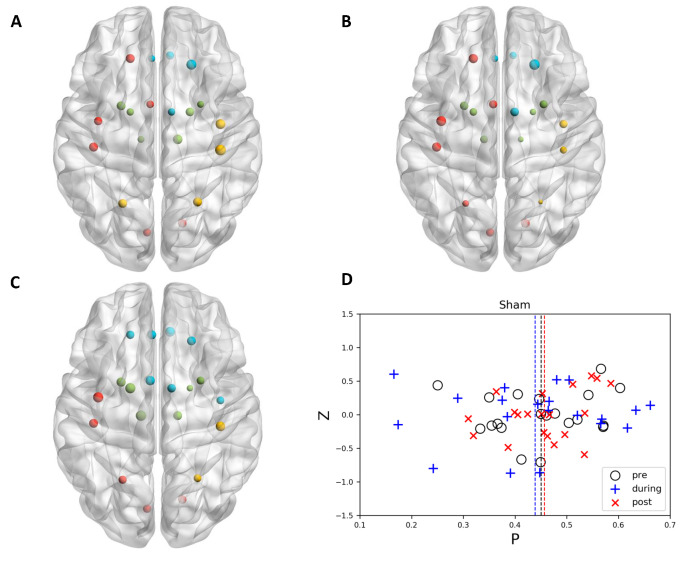
The node topology and community structure of sham stimulation in motor-related regions at (**A**) pre, (**B**) during, and (**C**) post time points (drawn by BrainNet Viewer [38].). Left orientation represents left side of the brain. Node size was determined by local efficiency. The nodes with the same color belonged to the same community in each subplot. (**D**) illustrated *P*-*z* plot for sham stimulation and the dashed line represents the mean *P* value for each time point.

**Figure 3:**
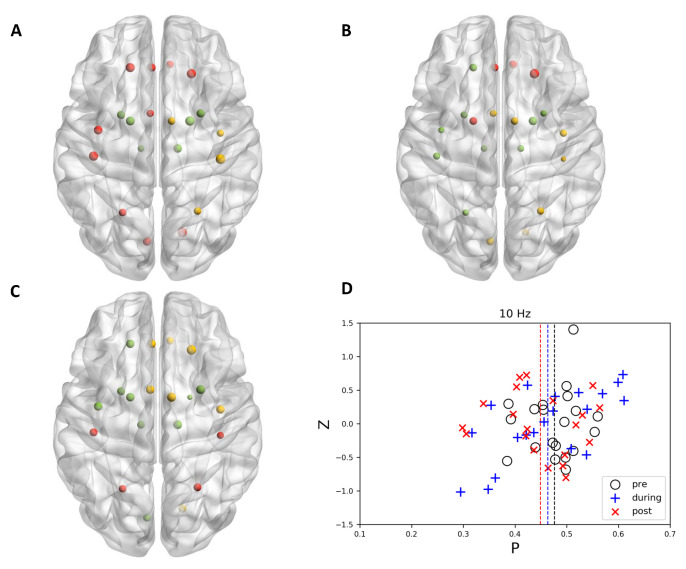
The node topology and community structure of 10 Hz stimulation in motor-related regions at (**A**) pre, (**B**) during, and (**C**) post time points. Left orientation represents left side of the brain. Panel (**D**) illustrated *P*-*z* plot for 10 Hz stimulation.

**Figure 4:**
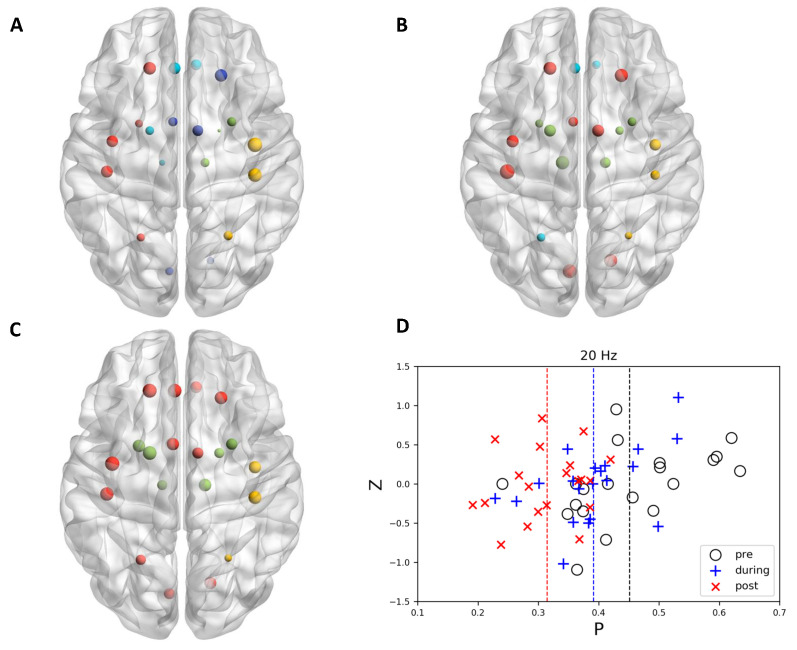
The node topology and community structure of 20 Hz stimulation in motor-related regions at (**A**) pre, (**B**) during, and (**C**) post time points. Left orientation represents left side of the brain. Panel (**D**) illustrated *P*-*z* plot for 20 Hz stimulation.

When no stimulation (sham) applied to the brain, it could be observed that the community structure and node affiliation to specific functional modules did not change significantly along with time (Figure 2). Different from sham stimulation, there existed evidence showing that 10 Hz stimulation tended to uniformly distribute the nodes to different communities. In some specific regions, the nodes that belong to various communities became more miscellaneous (Figure 3). Interestingly, opposite to 10 Hz stimulation, 20 Hz stimulation showed the ability to merge sub-modules into a larger community. All of the nodes in the same community dominantly located in one specific region and the space encompassed by nodes of different communities scarcely overlap with each other after 20 Hz stimulation (Figure 4).

### 3.2. Graph Theoretically Nodal Measures

It is often assumed that the role of a node could be determined by its position in the *P*-*z* parameter plane, which is called *P*-*z* plot [34]. Hence, we investigated the change of distributions of within-module degree z score and participation coefficient, respectively. From the *P*-*z* plots illustrated in Figure 2, Figure 3 and Figure 4, it could be observed that, for within-module degree z-score, the distributions did not show significant fluctuations, and the mean values of all conditions were located around zero. The repeated measure ANOVA also did not show any significant effect in within-module degree z-score. Post−hoc tests also indicated that no significant difference was observed for pairwise comparison.

**Figure 5:**
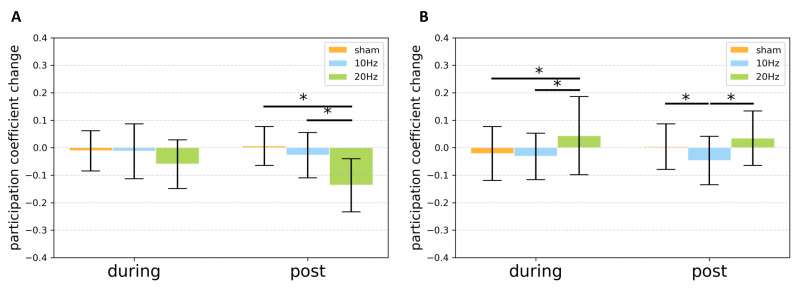
The bar chart of participation coefficient change of brain networks (**A**) in motor-related regions as well as (**B**) at the whole-brain level for various conditions during and after stimulation. Error bar stands for the standard error. Asterisk (*) indicates that a significant difference was observed at p< 0.05.

However, it is observed that, in 20 Hz stimulation, the distribution of participation coefficient shifted to a small value along with time. The repeated measure ANOVA of participation coefficient change indicated significant stimulation−time interaction effect (*F*(2, 38) = 5.527, p< 0.008). Post−hoc tests indicated a significant difference between sham and 20 Hz (p< 0.001, Bonferroni corrected) as well as 10 Hz and 20 Hz (p< 0.020, Bonferroni corrected) after stimulation (Figure 5A).

**Figure 6:**
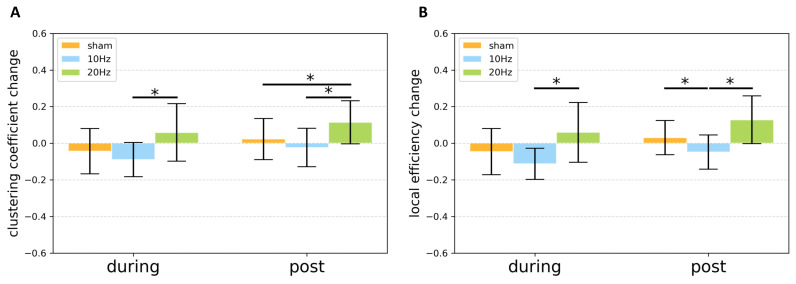
The bar chart of (**A**) clustering coefficient change and (**B**) local efficiency change of the network in motor-related regions for various conditions during and after stimulation. Error bar stands for the standard error. Asterisk (*) indicates that a significant difference was observed at p< 0.05.

For clustering coefficient change, the repeated measure ANOVA showed no significant stimulation−time interaction effect (*F*(2, 38) = 0.092, p< 0.912), but indicated a significant stimulation main effect (*F*(2, 38) = 10.294, p< 0.001). During stimulation, Post−hoc tests indicated a significant difference between 10 Hz and 20 Hz (p< 0.013, Bonferroni corrected). After stimulation, Post−hoc tests indicated a significant difference between sham and 20 Hz (p< 0.047, Bonferroni corrected) as well as between 10 Hz and 20 Hz (p< 0.027, Bonferroni corrected) (Figure 6A). Similarly, for local efficiency change, the repeated measure ANOVA also showed no significant stimulation−time interaction effect (*F*(2, 38) = 0.081, p< 0.923), but indicated significant stimulation main effect (*F*(2, 38) = 14.174, p< 0.001). During stimulation, the Post−hoc tests indicated a significant difference between 10 Hz and 20 Hz (p< 0.005, Bonferroni corrected). After stimulation, the Post−hoc tests indicated a significant difference between sham and 10 Hz (p< 0.007, Bonferroni corrected), as well as between 10 Hz and 20 Hz (p< 0.005, Bonferroni corrected) (Figure 6B).

Additionally, the modulation of the modular organization from the whole-brain network was also investigated. The repeated measure ANOVA of participation coefficient change indicated a significant stimulation−time interaction effect (*F*(2, 230) = 3.536, p< 0.035). During stimulation, Post−hoc tests indicated a significant difference between sham and 20 Hz (p< 0.004, Bonferroni corrected) as well as 10 Hz and 20 Hz (p< 0.001, Bonferroni corrected). After stimulation, Post−hoc tests indicated a significant difference between sham and 10 Hz (p< 0.001, Bonferroni corrected), as well as between 10 Hz and 20 Hz (p< 0.001, Bonferroni corrected) (Figure 5B). For a comprehensive understanding, the results of clustering coefficient and local efficiency analysis at the whole-brain level were also provided in the Supplementary Materials (Figure S2).

## 4. Discussion

This study aimed to investigate the tACS stimulation effect with 10 Hz and 20 Hz frequencies being applied in chronic stroke subjects using resting-state fMRI from a graph theoretical perspective. The results showed differential modulations induced by tACS with various frequencies. Meanwhile, the difference was also observed from brain networks in motor-related regions and whole-brain level, respectively. Our findings might facilitate effectively designing stimulation protocols with tACS in chronic stroke.

Evidence has accumulated that brain oscillations play an essential role in normal functioning through modulating the timing of neuronal spiking at the microscale and synchronizing distributed related cortical regions at the macroscale [39,40]. Specifically, the oscillations in alpha and beta bands were important and widely investigated by researchers. During relaxed alert states, alpha oscillations are supposed to be the most pronounced across most of the brain regions [41], and its functions were speculated to be involved in some aspects of attention and sensory processing [42,43]. Beta oscillations, especially those in sensorimotor brain regions, were usually motor-related and linked to activities, including motor observation, imagery, and execution [44]. In this context, non-invasive tACS has emerged as a powerful tool to modulate the brain activities and the internal brain states via entrainment of intrinsic frequency-specific oscillations [6,7]. In our study, the significant decrease of participation coefficient that indicated a higher segregation of communities was observed in motor-related regions after 20 Hz tACS, but no such effect was induced by 10 Hz tACS. In an age-related study, it has been exhibited that the participation coefficient was increased in older as compared with younger participants in the somatomotor networks probably due to less efficient use of neural resources [45]. This implied that 20 Hz tACS might be able to improve the cost efficiency of neural resources and make functional modules more differential and specific in the motor system. It has also been proposed that beta-band activity might correspond to an idling rhythm in the motor system [46] and allow for more efficient processing of feedback [47]. Hereby, the entrainment of beta oscillation after 20 Hz tACS might facilitate such processing by assembling sub-modules with higher functional coupling in motor-related regions.

It has been suggested that brain oscillations in different frequency ranges might enable regional interactions at different spatial scales [48]. Previous modeling studies implied that alpha and beta oscillations might support functional coupling over long distances [49]. Hence, the stimulation effect of tACS was also expected to be observed from the whole-brain perspective, although the stimulation site was located at the primary motor cortex. Our results illustrated that, at the whole-brain level, 10 Hz tACS facilitated segregation and 20 Hz facilitated the integration of communities. It has been revealed that the presence of alpha generators existed across all cortical layers [50] and similar alpha physiology was found across the whole brain, which implied an integrative function of alpha wave, especially under the resting condition [51]. Different from alpha oscillation, the function of beta frequency in the whole-brain level was not explicit. In our study, it seemed that, the communication of whole-brain communities, was enhanced after 20 Hz tACS, characterized by an increase in the participation coefficient. This could be partially explained by the findings in previous studies, which exhibited that beta oscillations are ideally suited for communicating across long conduction delays [49,52,53].

Additionally, since the stimulation site was located over the primary motor cortex, we were also interested in the modulation effect of nodal properties in the motor system. Hereby, the nodal measures of clustering coefficient and local efficiency, which shared similar meaning, were adopted. For both local measures, the increasing trend was observed after 20 Hz stimulation in motor-related regions, but the only decreasing trend was observed after 10 Hz stimulation. This implied that 20 Hz tACS might improve the efficiency of information transmission within specific modules and such an effect could maintain after stimulation [18]. Opposite with 20 Hz tACS, 10 Hz tCAS led to decreased local efficiency which might indicate a pruning of task-irrelevant connections [54]. Together, it suggested that the motor system might show frequency-specific responses to extrinsic stimulation and be prone to be more sensitive to entrainment of beta-band oscillations.

It is also worth noting that, although some previous studies have exhibited the modulation effect of tACS with different frequencies on healthy participants, few studies have investigated the influence of tACS in stroke patients [12,20,55,56]. One study claimed that tACS might facilitate lesioned brain self-regulation during neurofeedback intervention [57]. Our study tried to uncover the modulation effect of frequency-specific tACS in the chronic stroke from the view of brain networks with fMRI data at the same time. On the other hand, it has been proposed that the human brain always seeks a balance between the local segregation of function and the global integration of information [58]. Based on our findings, 20 Hz might have the potential to assist the lesioned brain to reach such an optimal state and it could be a promising tool applied in routine rehabilitation therapies. However, randomized controlled trials were needed in order to verify this point and determine the frequency that can maximally accelerate the recovery process for stroke patients in the future. In the current study, the investigation of the effect on motor function and motor learning after the single-session tACS could not be evidenced by the results directly and was quite limited due to the lack of behavioral assessment. Some previous studies have indicated that a single-session tDCS could help chronic stroke subjects to shorten the response time of tasks and improved pinch force in the paretic hand [59,60]. Lefebvre et al. suggested that a single-session dual-tDCS could enhance online motor skill learning and facilitate precision grip as well as dexterity for chronic stroke patients [61,62]. Different from tDCS, the acute effect of a single-session tACS was merely investigated. Hence, the experimental design could be improved by collecting some behavioral data before and after the stimulation to better understand this point in the future.

Several limitations should be noted in our study. The network status of the stroke subjects may be different due to the various lesions in sizes and locations. In the present study, we did not take the lesion information into account, because most subjects had relatively homogeneous lesions in sizes and locations. Meanwhile, we adopted the repeated measures design (each subject underwent all stimulation conditions) which could control for factors that cause variability between subjects. In this way, the influence of network status variations that are caused by lesions could be reduced to some extent. However, to make the results more precise, in the future subjects with sufficiently homogeneous neural injury should be recruited and more advanced analytical methods that take the lesion formation into account should be utilized. On the other hand, to make the findings more valid, it is better to check the difference in the node topography between stroke patients and healthy adults. Caution should be taken when interpreting our findings due to the lack of such comparison in the present study. Besides, it is really inevitable that there are multiple co-existing states when resting as well as diversity among stroke subjects in different experimental sessions, which might introduce some variations in the pre-stimulation community structures. Therefore, due to the existing variability of community structures in the baseline, precaution should be taken while qualitatively interpreting these observations. Although we proposed to mainly stimulate the primary motor area, the current montage with huge stimulation pads might lead to the diffusion of stimulation effect. Hence, a high-definition tACS with the centering montage [56] could be adopted to be more specific in the future. Besides, the sample size was not large, which might limit the generalization power to some extent. More patients should be recruited to validate and extend the findings of the current study.

## 5. Conclusions

In summary, we investigated the frequency-specific stimulation effect of tACS in chronic stroke while using resting-state fMRI data. The graph theoretical analysis mainly indicated the differential modulation effect of network integration and segregation properties in motor-related regions as well as at the whole-brain level after 10 Hz and 20 Hz tACS intervention. This study might facilitate designing neurorehabilitation protocols with tACS for stroke survivors in the future.

## Data Availability

The data presented in this study are available on proper request from the corresponding author.

## Supplementary Materials

The following are available online at https://www.mdpi.com/2076-3425/11/3/377/s1, Figure S1: Lesion distribution of stroke subjects, Figure S2: The bar chart of clustering coefficient change and local efficiency change of the network at the whole-brain level for various conditions during and after stimulation, Table S1: Demographics and clinical properties of the participants, Table S2: AAL ROIs in motor-related regions.

## References

[1] Stinear C., Lang C., Zeiler S., Byblow W. (2020). Advances and challenges in stroke rehabilitation. Lancet Neurol..

[2] Jäncke L. (2009). The plastic human brain. Restor. Neurol. Neurosci..

[3] Norouzi-Gheidari N., Archambault P., Fung J. (2012). Effects of robot-assisted therapy on stroke rehabilitation in upper limbs: Systematic review and meta-analysis of the literature. J. Rehabil. Res. Dev..

[4] Duret C., Grosmaire A.G., Krebs H. (2019). Robot-Assisted Therapy in Upper Extremity Hemiparesis: Overview of an Evidence-Based Approach. Front. Neurol..

[5] Bao S.C., Khan A., Song R., Tong R.K.Y. (2020). Rewiring the Lesioned Brain: Electrical Stimulation for Post-Stroke Motor Restoration. J. Stroke.

[6] Purpura D., McMurtry J. (1965). Intacellular activities and evoked potential changes during polarization of motor cortex. J. Neurophysiol..

[7] Nitsche M., Paulus W. (2000). Excitability changes induced in the human motor cortex by weak transcranial direct current stimulation. J. Physiol..

[8] Thut G., Schyns P., Gross J. (2011). Entrainment of Perceptually Relevant Brain Oscillations by Non-Invasive Rhythmic Stimulation of the Human Brain. Front. Psychol..

[9] Schnitzler A., Gross J. (2005). Normal and pathological oscillatory communication in the brain. Nat. Rev. Neurosci..

[10] Salmelin R., Hari R. (1994). Characterization of spontaneous MEG rhythms in healthy adults. Electroencephalogr. Clin. Neurophysiol..

[11] Feurra M., Blagoveshchensky E., Nikulin V., Nazarova M., Lebedeva A., Pozdeeva D., Yurevich M., Rossi S. (2019). State-Dependent Effects of Transcranial Oscillatory Currents on the Motor System during Action Observation. Sci. Rep..

[12] Pollok B., Boysen A.C., Krause V. (2015). The effect of transcranial alternating current stimulation (tACS) at alpha and beta frequency on motor learning. Behav. Brain Res..

[13] Meier A., Krause V., Pollok B. (2014). Early motor memory consolidation: Effects of 10 Hz and 20 Hz transcranial alternating current stimulation (tACS) over the left primary motor cortex (M1). Klin. Neurophysiol..

[14] Kwon Y.H., Ko M.H., Ahn S., Kim Y.H., Song J., Lee C.H., Chang M., Jang S. (2008). Primary motor cortex activation by transcranial direct current stimulation in the human brain. Neurosci. Lett..

[15] Ali M., Sellers K., Frohlich F. (2013). Transcranial Alternating Current Stimulation Modulates Large-Scale Cortical Network Activity by Network Resonance. J. Neurosci. Off. J. Soc. Neurosci..

[16] Kimberley T., Khandekar G., Borich M. (2008). fMRI reliability in subjects with stroke. Exp. Brain Res..

[17] Van den Heuvel M.P., Hulshoff Pol H.E. (2010). Exploring the brain network: A review on resting-state fMRI functional connectivity. Eur. Neuropsychopharmacol..

[18] Farahani F., Karwowski W., Lighthall N. (2019). Application of Graph Theory for Identifying Connectivity Patterns in Human Brain Networks: A Systematic Review. Front. Neurosci..

[19] Solomons C. (2019). A Review of Transcranial Electrical Stimulation Methods in Stroke Rehabilitation. Neurol. India.

[20] Wach C., Krause V., Moliadze V., Paulus W., Schnitzler A., Pollok B. (2012). Effects of 10 Hz and 20 Hz transcranial alternating current stimulation (tACS) on motor functions and motor cortical excitability. Behav. Brain Res..

[21] Wang Y., Shi L., Dong G., Zhang Z., Chen R. (2020). Effects of Transcranial Electrical Stimulation on Human Auditory Processing and Behavior—A Review. Brain Sci..

[22] Thielscher A., Antunes A., Saturnino G. Field modeling for transcranial magnetic stimulation: A useful tool to understand the physiological effects of TMS?. Proceedings of the 2015 37th Annual International Conference of the IEEE Engineering in Medicine and Biology Society (EMBC).

[23] Yan C.G., Zang Y.F. (2010). DPARSF: A MatLab toolbox for “pipeline” data analysis of resting-state fMRI. Front. Syst. Neurosci..

[24] Friston K., Williams S., Howard R., Frackowiak R., Turner R. (1996). Movement-Related effects in fMRI time-series. Magn. Reson. Med..

[25] Power J., Barnes K., Snyder A., Schlaggar B., Petersen S. (2012). Spurious but systematic conditions in functional connectivity MRI networks arise from subject motion. Neuroimage.

[26] Zuo X.N., Di Martino A., Kelly C., Shehzad Z., Gee D., Klein D., Castellanos F., Biswal B., Milham M. (2009). The Oscillating Brain: Complex and Reliable. NeuroImage.

[27] Tzourio-Mazoyer N., Landeau B., Papathanassiou D., Crivello F., Etard O., Delcroix N., Mazoyer B., Joliot M. (2002). Automated Anatomical Labeling of Activations in SPM Using a Macroscopic Anatomical Parcellation of the MNI MRI Single-Subject Brain. NeuroImage.

[28] Bear M., Connors B., Paradiso M. (2015). Neuroscience: Exploring the Brain.

[29] Cambridge U., Cohen P. (1983). Applied Multiple Regression/Correlation Analysis for The Behavioral Sciences. Am. J. Cardiol..

[30] Fornito A., Zalesky A., Bullmore E. (2010). Network Scaling Effects in Graph Analytic Studies of Human Resting-State fMRI Data. Front. Syst. Neurosci..

[31] Garrison K., Scheinost D., Finn E., Shen X., Constable R. (2015). The (in)stability of functional brain network measures across thresholds. NeuroImage.

[32] Rubinov M., Sporns O. (2010). Complex network measures of brain connectivity: Uses and interpretations. NeuroImage.

[33] Newman M. (2004). Fast algorithm for detecting community structure in networks. Phys. Rev. E Stat. Nonlinear Soft Matter Phys..

[34] Guimerà R., Amaral L. (2005). Functional Cartography of Complex Metabolic Networks. Nature.

[35] Guimerà R., Amaral L. (2005). Cartography of complex networks: Modules and universal roles. J. Stat. Mech..

[36] Watts D., Strogatz S. (2011). Collective dynamics of ’small-world’ networks. Nature.

[37] Latora V., Marchiori M. (2001). Efficient Behavior of Small-World Networks. Phys. Rev. Lett..

[38] Xia M., Wang J., He Y. (2013). BrainNet Viewer: A Network Visualization Tool for Human Brain Connectomics. PLoS ONE.

[39] Jacobs J., Kahana M., Ekstrom A., Fried I. (2007). Brain Oscillations Control Timing of Single-Neuron Activity in Humans. J. Neurosci. Off. J. Soc. Neurosci..

[40] Fries P. (2001). A mechanism for cognitive dynamics: Neuronal communication through neuronal coherence. Trends Cogn. Sci..

[41] Srinivasan R., Winter W., Nunez P. (2006). Source analysis of EEG oscillations using high-resolution EEG and MEG. Prog. Brain Res..

[42] Voytek B., Canolty R., Shestyuk A., Crone N., Parvizi J., Knight R. (2010). Shifts in Gamma Phase–Amplitude Coupling Frequency from Theta to Alpha Over Posterior Cortex During Visual Tasks. Front. Hum. Neurosci..

[43] Jensen O., Mazaheri A. (2010). Shaping Functional Architecture by Oscillatory Alpha Activity: Gating by Inhibition. Front. Hum. Neurosci..

[44] Honaga E., Ishii R., Kurimoto R., Ikezawa K., Takahashi H., Nakahachi T., Iwase M., Mizuta I., Yoshimine T., Takeda M. (2010). Post-movement beta rebound abnormality as indicator of mirror neuron system dysfunction in autistic spectrum disorder: An MEG study. Neurosci. Lett..

[45] Geerligs L., Renken R., Saliasi E., Maurits N., Lorist M. (2014). A Brain-Wide Study of Age-Related Changes in Functional Connectivity. Cereb. Cortex.

[46] Pfurtscheller G., Stancak A., Neuper C. (1996). Post-movement beta synchronization. A correlate of an idle motor area?. Electroencephalogr. Clin. Neurophysiol..

[47] Baker S. (2008). Oscillatory interactions between sensorimotor cortex and the periphery. Curr. Opin. Neurobiol..

[48] Rosanova M., Casali A., Bellina V., Resta F., Mariotti M., Massimini M. (2009). Natural Frequencies of Human Corticothalamic Circuits. J. Neurosci. Off. J. Soc. Neurosci..

[49] Kopell N., Ermentrout B., Whittington M., Traub R. (2000). Gamma rhythms and beta rhythms have different synchronization properties. Proc. Natl. Acad. Sci. USA.

[50] Haegens S., Barczak A., Musacchia G., Lipton M., Mehta A., Lakatos P., Schroeder C. (2015). Laminar Profile and Physiology of the Rhythm in Primary Visual, Auditory, and Somatosensory Regions of Neocortex. J. Neurosci..

[51] Halgren M., Ulbert I., Bastuji H., Fabó D., Eross L., Rey M., Devinsky O., Doyle W., Mak-McCully R., Halgren E. (2019). The generation and propagation of the human alpha rhythm. Proc. Natl. Acad. Sci. USA.

[52] Groppe D., Bickel S., Keller C., Jain S., Hwang S., Harden C., Mehta A. (2013). Dominant frequencies of resting human brain activity as measured by the electrocorticogram. NeuroImage.

[53] Bibbig A., Traub R., Whittington M. (2002). Long-range synchronization of *γ* and *β* oscillations and the plasticity of excitatory and inhibitory synapses: A network model. J. Neurophysiol..

[54] Cohen J., D’Esposito M. (2016). The Segregation and Integration of Distinct Brain Networks and Their Relationship to Cognition. J. Neurosci..

[55] Feurra M., Pasqualetti P., Bianco G., Santarnecchi E., Rossi A., Rossi S. (2013). State-Dependent Effects of Transcranial Oscillatory Currents on the Motor System: What You Think Matters. J. Neurosci. Off. J. Soc. Neurosci..

[56] Heise K.F., Kortzorg N., Saturnino G., Fujiyama H., Cuypers K., Thielscher A., Swinnen S. (2016). Evaluation of a Modified High-Definition Electrode Montage for Transcranial Alternating Current Stimulation (tACS) of Pre-Central Areas. Brain Stimul..

[57] Naros G., Gharabaghi A. (2017). Physiological and behavioral effects of *β*-tACS on brain self-regulation in chronic stroke. Brain Stimul..

[58] Lord L.D., Stevner A.B., Deco G., Kringelbach M.L. (2017). Understanding principles of integration and segregation using whole-brain computational connectomics: Implications for neuropsychiatric disorders. Philos. Trans. R. Soc. A Math. Phys. Eng. Sci..

[59] Hummel F., Voller B., Celnik P., Floel A., Giraux P., Gerloff C., Cohen L. (2006). Effects of brain polarization on reaction times and pinch force in chronic stroke. BMC Neurosci..

[60] Stagg C., Bachtiar V., O’Shea J., Allman C., Bosnell R., Kischka U., Matthews P., Johansen-Berg H. (2011). Cortical activation changes underlying stimulation induced behavioral gains in chronic stroke. Brain J. Neurol..

[61] Lefebvre S., Laloux P., Peeters A., Desfontaines P., Jamart J., Vandermeeren Y. (2013). Dual-tDCS Enhances Online Motor Skill Learning and Long-Term Retention in Chronic Stroke Patients. Front. Hum. Neurosci..

[62] Lefebvre S., Thonnard J.L., Laloux P., Peeters A., Jamart J., Vandermeeren Y. (2013). Single Session of Dual-tDCS Transiently Improves Precision Grip and Dexterity of the Paretic Hand After Stroke. Neurorehabilit. Neural Repair.

