# Supplementary material for: The Effects of 10 Hz and 20 Hz tACS in Network Integration and Segregation in Chronic Stroke: A Graph Theoretical fMRI Study
Source: Brain Sci. 2021 Mar 16;11(3):377. doi: 10.3390/brainsci11030377 (PMC8002277; doi:10.3390/brainsci11030377)
Supplement: Supplementary file 1 [file brainsci-11-00377-s001.pdf]

Supplementary Materials: The effects of 10Hz and 20Hz tACS in network integration and segregation in chronic stroke: A graph theoretical fMRI study

Cheng Chen, Kai Yuan, Winnie Chiu-wing Chu and Raymond Kai-yu Tong

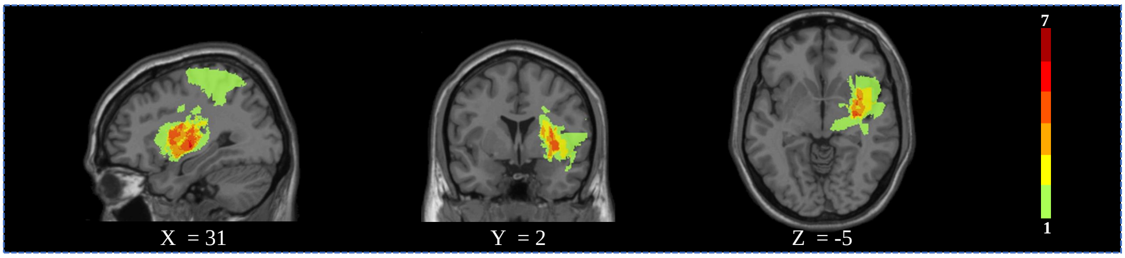

Figure S1. Lesion distribution of stroke subjects. The color bar represents the number of patients with lesions in the corresponding areas. The annotation of X, Y, Z represented the coordinate of the slice in MNI space. In second and third plots, left orientation represents left side of the brain.

Table S1. Demographics and clinical properties of the participants.

Subject	Gender	Age	Stroke onset (y)	Stroke type	Lesion side	FMA	ARAT
S1	Male	65-69	0.7	Ischemic	Left	4	0
S2	Male	50-54	3	Ischemic	Right	15	11
S3	Female	65-69	2	Ischemic	Left	17	0
S4	Male	50-54	1	Ischemic	Left	32	6
S5	Female	70-74	9	Ischemic	Left	22	17
S6	Male	55-59	3	Ischemic	Left	55	57
S7	Female	60-64	3	Ischemic	Left	47	53
S8	Male	55-59	4	Ischemic	Right	49	54
S9	Male	55-59	4	Ischemic	Right	27	23
S10	Female	70-74	1	Ischemic	Right	8	9
S11	Female	70-74	6	Ischemic	Right	45	51
S12	Male	60-64	3	Hemorrhage	Right	24	22
S13	Male	35-39	3	Hemorrhage	Right	27	8
mean±std		61±10				29±16	24±21

Abbreviations: FMA = Fugel-Meyer Assessment for upper limb; ARAT = Action Research Arm Test.

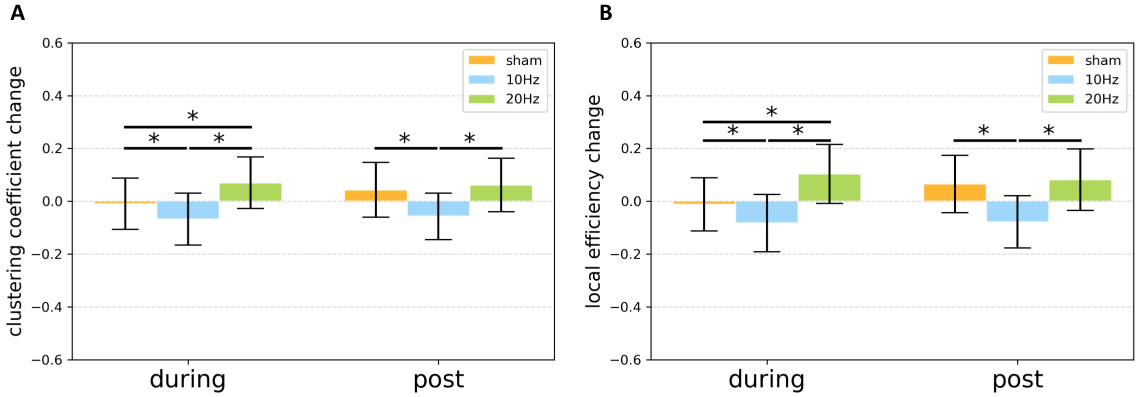

Figure S2. The bar chart of (A) clustering coefficient change and (B) local efficiency change of the network at the whole-brain level for various conditions during and after stimulation. Error bar stands for the standard error. Asterisk (*) indicates that a significant difference was observed at $p < 0.05$.

Clustering coefficient and local efficiency analysis at the whole-brain level

For clustering coefficient change at the whole-brain level, the repeated measure ANOVA showed significant $stimulation - time$ interaction effect (F(2, 230)=13.025, $p < 0.001$). During stimulation, $Post - hoc$ tests indicated a significant difference between $sham$ and $10Hz$, $sham$ and $20Hz$, as well as $10Hz$ and $20Hz$ (all $p < 0.001$, Bonferroni corrected). After stimulation, $Post - hoc$ tests indicated a significant difference between $sham$ and $10Hz$ as well as $10Hz$ and $20Hz$ (both $p < 0.001$, Bonferroni corrected) (Figure S2A). Similarly, for local efficiency change at the whole-brain level, the repeated measure ANOVA showed significant $stimulation - time$ interaction effect (F(2, 230)=30.285, $p < 0.001$). During stimulation, $Post - hoc$ tests indicated a significant difference between $sham$ and $10Hz$, $sham$ and $20Hz$, as well as $10Hz$ and $20Hz$ (all $p < 0.001$, Bonferroni corrected). After stimulation, $Post - hoc$ tests indicated a significant difference between $sham$ and $10Hz$ as well as $10Hz$ and $20Hz$ (both $p < 0.001$, Bonferroni corrected) (Figure S2B). These results also illustrated the heterogeneous modulation effects of 10Hz and 20Hz tACS. Interestingly, it could be observed that both 10Hz and 20Hz tACS showed a slightly different modulation behaviour between in motor-related regions and at the whole-brain level, compared with the results of corresponding analysis in motor-related regions (Figure 6). This phenomenon could also be found in network integration and segregation properties, which implied that the modulation effect of a specific stimulation might be different if observed from different networks.

Table S2. AAL ROIs in motor-related regions.

			MNI coordinates		
**Index**	**L (R) side**	**abbreviation**	**X**	**Y**	**Z**
1	L	Precentral_L	-39	-6	51
2	R	Precentral_R	41	-8	52
3	L	Frontal_Sup_L	-18	35	42
4	R	Frontal_Sup_R	22	31	44
19	L	Supp_Motor_Area_L	-5	5	61
20	R	Supp_Motor_Area_R	9	0	62
31	L	Cingulum_Ant_L	-4	35	14
32	R	Cingulum_Ant_R	8	37	16
43	L	Calcarine_L	-7	-79	6
44	R	Calcarine_R	16	-73	9
57	L	Postcentral_L	-42	-23	49
58	R	Postcentral_R	41	-25	53
59	L	Parietal_Sup_L	-23	-60	59
60	R	Parietal_Sup_R	26	-59	62
73	L	Putamen_L	-24	4	2
74	R	Putamen_R	28	5	2
75	L	Pallidum_L	-18	0	0
76	R	Pallidum_R	21	0	0
77	L	Thalamus_L	-11	-18	8
78	R	Thalamus_R	13	-18	8
